# Data for amino acid alignment of Japanese stingray melanocortin receptors with other gnathostome melanocortin receptor sequences, and the ligand selectivity of Japanese stingray melanocortin receptors

**DOI:** 10.1016/j.dib.2016.04.050

**Published:** 2016-04-26

**Authors:** Akiyoshi Takahashi, Perry Davis, Christina Reinick, Kanta Mizusawa, Tatsuya Sakamoto, Robert M. Dores

**Affiliations:** a School of Marine Biosciences, Kitasato Universtiy, Sagaminara 252-0373, Japan; b Department of Biological Sciences, University of Denver, Denver, CO 80210, USA; c Ushimado Marine Institute, Faculty of Sciences, Okayama University, Ushimado, Setouchi 701-4303, Japan

**Keywords:** Adrenocorticotropic hormone (ACTH), *Dasyatis akajei*, Melanocortin receptor (MCR), Melanocyte-stimulating hormone (MSH), Stingray

## Abstract

This article contains structure and pharmacological characteristics of melanocortin receptors (MCRs) related to research published in “Characterization of melanocortin receptors from stingray *Dasyatis akajei*, a cartilaginous fish” (Takahashi et al., 2016) [1]. The amino acid sequences of the stingray, *D. akajei*, MC1R, MC2R, MC3R, MC4R, and MC5R were aligned with the corresponding melanocortin receptor sequences from the elephant shark, *Callorhinchus milii*, the dogfish, *Squalus acanthias*, the goldfish, *Carassius auratus*, and the mouse, *Mus musculus*. These alignments provide the basis for phylogenetic analysis of these gnathostome melanocortin receptor sequences. In addition, the Japanese stingray melanocortin receptors were separately expressed in Chinese Hamster Ovary cells, and stimulated with stingray ACTH, α-MSH, β-MSH, γ-MSH, δ-MSH, and β-endorphin. The dose response curves reveal the order of ligand selectivity for each stingray MCR.

**Specifications Table**

**Table:** 

Subject area	Biology
More specific subject area	Endocrinology
Type of data	Text files, graphs
How data was acquired	Amino acid sequences were aligned using MEGA 6.0. Ligand selectivity assays were done using the CRE/Luciferase reporter assay [2]. Luminescence was measured using a Bio-Tek Synergy HT plate reader (Bio Tek, Winooski, VT, USA), and the data were analyzed and graphed using Kaleidagraph software (Synergy Software, Reading, PA, USA)
Data format	Raw
Experimental factors	Melanocortin DNAs were cloned from stingray genomic DNA or brain mRNA. Cloned DNA were expressed in Chinese Hamster Ovary cells
Experimental features	Sequence alignment was done using MEGA 6.0. The ligand selectivity assays were done as described in reference [3].
Data source location	Kitasato University, Sagamihara, Kanagawa, Japan. University of Denver, Denver, Colorado, USA
Data accessibility	Data is within this article

**Value of the data**

• These data are valuable for researchers participated in endocrinology of primitive fish and evolution of melanocortin systems.
• These could be used as probes to explore orthologs in other cartilaginous fish such as skates, sharks and chimaeras.
• The data on ligand selectivity could be useful tools for structure–function relationship studies in endocrinology and pharmacology.

## 1 Data

**Fig. 1:**
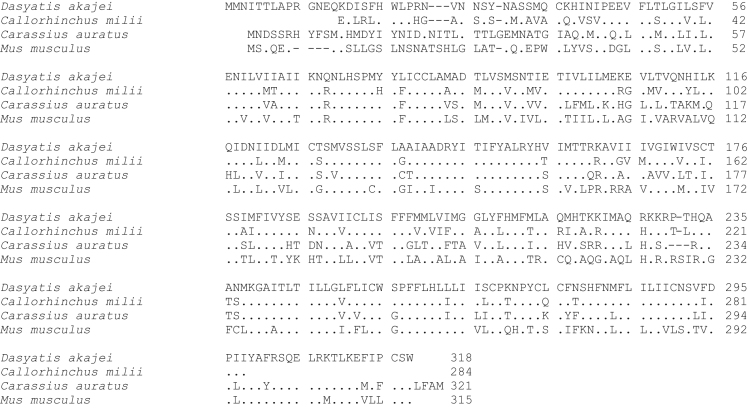
Amino acid sequence comparison of MC1R used for phylogenetic analysis. Species names are *Dasyatis akajei* for stingray, *Callorhinchus milii* for elephant shark, *Carassius auratus* for goldfish, and *Mus musculus* for mouse. Dot shows identical amino acid to stingray sequence. Hyphen shows gap. Accession numbers: LC108746 (*Dasyatis akajei*), BR000855 (*Callorhinchus milii*), AB618067 (*Carassius auratus*), and BC119296 (*Mus musculus*). The percent identify for the MC1R orthologs was 33%.

**Fig. 2:**
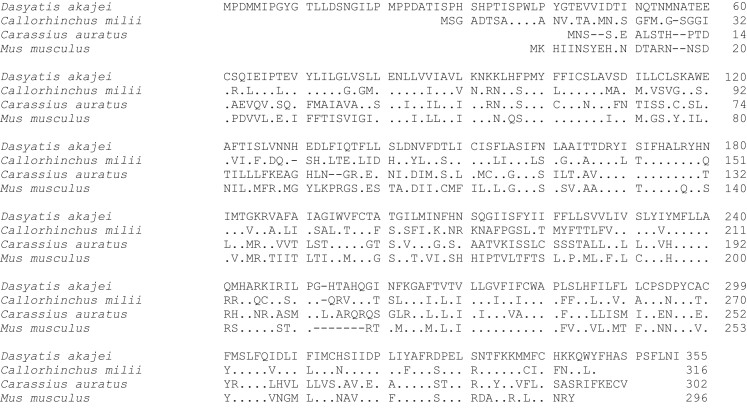
Amino acid sequence comparison of MC2R used for phylogenetic analysis. Species names are *Dasyatis akajei* for stingray, *Callorhinchus milii* for elephant shark, *Carassius auratus* for goldfish, and *Mus musculus* for mouse. Dot shows identical amino acid to stingray sequence. Hyphen shows gap. Accession numbers: LC108747 (*Dasyatis akajei*), BR000856 (*Callorhinchus milii*), AB618068 (*Carassius auratus*), and NM_008560 (*Mus musculus*). The percent identity for the MC2R orthologs was 24%.

**Fig. 3:**
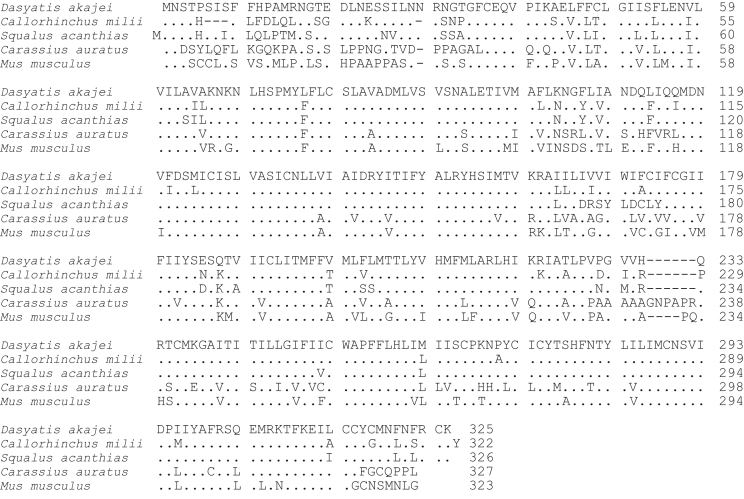
Amino acid sequence comparison of MC3R used for phylogenetic analysis. Species names are *Dasyatis akajei* for stingray, *Callorhinchus milii* for elephant shark, *Squalus acanthias* for dogfish, *Carassius auratus* for goldfish, and *Mus musculus* for mouse. Dot shows identical amino acid to stingray sequence. Hyphen shows gap. Accession numbers: LC108748 (*Dasyatis akajei*), BR000857 (*Callorhinchus milii*), AY560605 (*Squalus acanthias*), AB618069 (*Carassius auratus*), and NM_008561 (*Mus musculus*). The percent identity for the MC3R orthologs was 52%.

**Fig. 4:**
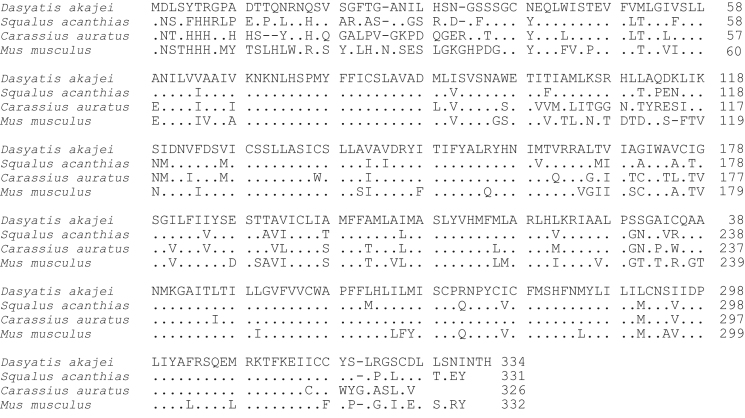
Amino acid sequence comparison of MC4R used for phylogenetic analysis. Species names are *Dasyatis akajei* for stingray, *Squalus acanthias* for dogfish, *Carassius auratus* for goldfish, and *Mus musculus* for mouse. Dot shows identical amino acid to stingray sequence. Hyphen shows gap. Accession numbers: LC108749 (*Dasyatis akajei*), AY169401 (*Squalus acanthias*), AJ534337 (*Carassius auratus*), and BC116959 (*Mus musculus*). The percent identity for the MC4R orthologs was 55%.

**Fig. 5:**
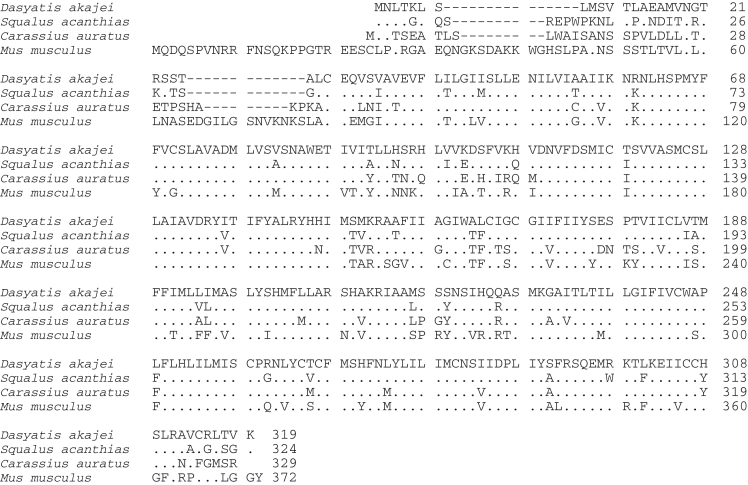
Amino acid sequence comparison of MC5R used for phylogenetic analysis. Species names are *Dasyatis akajei* for stingray, *Squalus acanthias* for dogfish, *Carassius auratus* for goldfish, and *Mus musculus* for mouse. Dot shows identical amino acid to stingray sequence. Hyphen shows gap. Accession numbers: LC108750 (*Dasyatis akajei*), AY562212 (*Squalus acanthias*), AJ576322 (*Carassius auratus*), and BC100720 (*Mus musculus*). The percent identity for the MC5R orthologs was 61%.

**Fig. 6:**
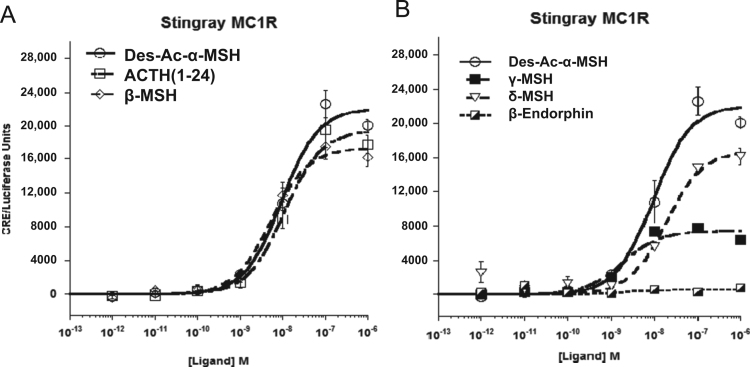
Ligand selectivity of stingray MC1R. (A) Functional activation of the stingray MC1R after stimulation with the following stingray melanocortins: Des-acetyl-α-MSH (Des-Ac-α-MSH), ACTH(1-24), or β-MSH. (B) Functional activation of stingray MC1R after stimulation with the following stingray melanocortins: Des-Ac-α-MSH, γ-MSH, δ-MSH or β-endorphin(1-20). As described in methods, CHO cells were transiently transfected with a stingray *mc*1*r* cDNA construct and a *cre*/*luc* cDNA construct. Two days post-transfection, wells containing 1×10^5^ cells were stimulated with the stingray melanocortin ligands at concentrations ranging from 10^−^^6^ M to 10^−^^12^ M. Results are expressed as mean±S.E.M.; *n*=3.

**Fig. 7:**
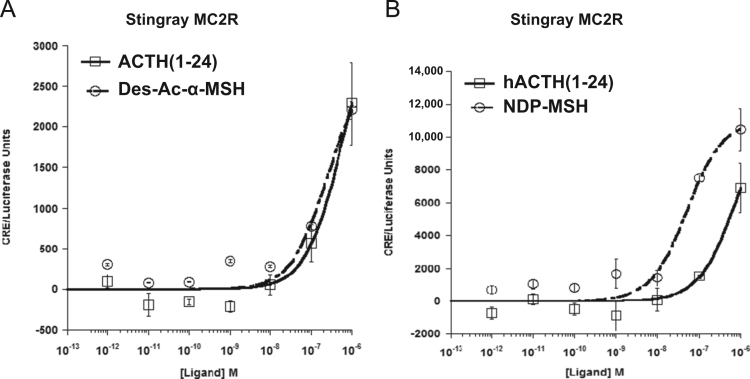
Ligand selectivity of stingray MC2R. (A) Functional activation of the stingray MC2R after stimulation with stingray Des-acetyl-α-MSH (Des-Ac-α-MSH) or stingray ACTH(1-24). (B) Functional activation of stingray MC2R after stimulation with human ACTH(1-24) (hACTH(1-24)) or NDP-MSH. The activation assays were performed as described in the figure legend for Fig. 6. Results are expressed as mean±S.E.M.; *n*=3.

**Fig. 8:**
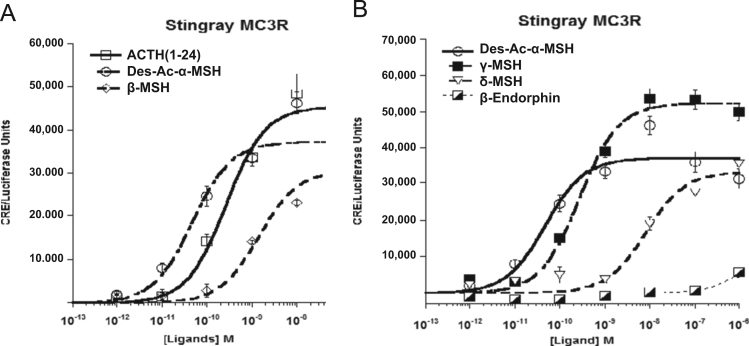
Ligand selectivity of stingray MC3R. (A) Functional activation of the stingray MC3R after stimulation with the following stingray melanocortins: Des-acetyl-α-MSH (Des-Ac-α-MSH), ACTH(1-24), or β-MSH. (B) Functional activation of the stingray MC3R after stimulation with the following stingray melanocortins: Des-acetyl-α-MSH (Des-Ac-α-MSH), γ-MSH, δ-MSH or β-endorphin(1-20). The activation assays were performed as described in the figure legend for Fig. 6. Results are expressed as mean±S.E.M.; *n*=3.

**Fig. 9:**
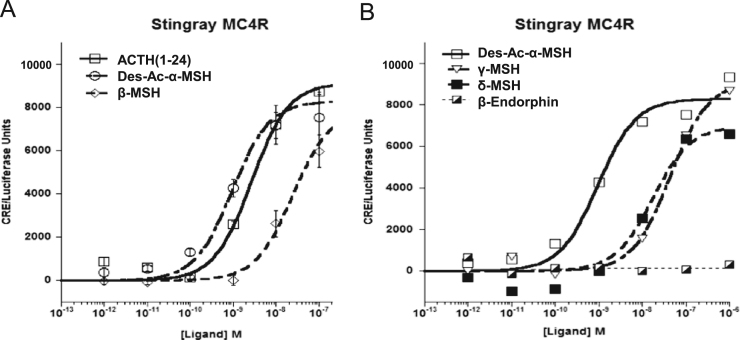
Ligand selectivity of stingray MC4R. (A) Functional activation of the stingray MC4R after stimulation with the following stingray melanocortins: Des-acetyl-α-MSH (Des-Ac-α-MSH), ACTH(1-24), or β-MSH. (B) Functional activation of stingray MC4R after stimulation with the following stingray melanocortins: Des-acetyl-α-MSH (Des-Ac-α-MSH), γ-MSH, δ-MSH or β-endorphin(1-20). The activation assays were performed as described in the figure legend for Fig. 6. Results are expressed as mean±S.E.M.; *n*=3.

**Fig. 10:**
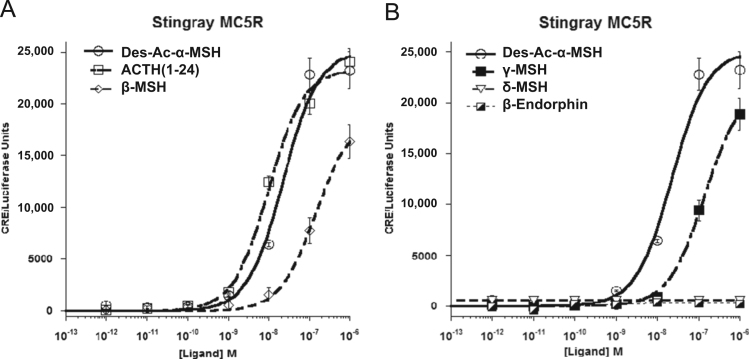
Ligand selectivity of stingray MC5R. (A) Functional activation of the stingray MC5R after stimulation with the following stingray melanocortins: Des-acetyl-α-MSH (Des-Ac-α-MSH), ACTH(1-24), or β-MSH. (B) Functional activation of stingray MC5R after stimulation with the following stingray melanocortins: Des-acetyl-α-MSH (Des-Ac-α-MSH), γ-MSH, δ-MSH or β-endorphin(1-20). The activation assays were performed as described in the figure legend for Fig. 6. Results are expressed as mean±S.E.M.; *n*=3.

Data provided in this article show amino acid sequence comparison of melanocortin receptors (MCRs) in vertebrates and ligand selectivity of stingray MC peptides on these receptors. The amino acids sequences of MC1R (Fig. 1), MC2R (Fig. 2), MC3R (Fig. 3), MC4R (Fig. 4), and MC5R (Fig. 5) of stingray (*Squalus acanthias*) which determined by us [1] were compared to corresponding sequences from two species of other cartilaginous fishes (i.e., *Callorhinchus milii*, elephant shark and *S. acanthias*, dogfish), a teleost (*Carassius auratus*, goldfish), and a mammal (*Mus musculus*, mouse). Data are also provided for ligand selectivity include effects of stingray Des-acetyl-α-MSH, β-MSH, γ-MSH, δ-MSH, ACTH(1-24) and β-endorphin on MC1R, MC3R, MC4R, and MC5R (Fig. 6, Fig. 8, Fig. 9, Fig. 10) and those of stingray Des-acetyl-α-MSH, ACTH(1-24), human ACTH(1-24) and NDP-MSH on stingray MC2R (Fig. 7).

## 2 Experimental design, materials and methods

In order to align the amino acid sequences of the melanocortin receptors for the Japanese stingray, *D. akajei*, the dogfish, *S. acanthias,* the elephant shark, *C. milii*, the goldfish, *C. auratus*, and the mouse, *M. musculus*, it was essential to identify putative transmembrane domains in each receptor sequence. To this end, the program ‘‘MEMSAT3’’ (http://bioinf.cs.ucl.ac.uk/psipred/) was used. The amino acid sequences where then aligned using the program MEGA 6.0.

To functionally express and determine the ligand selectivity of the stingray (sr) MC1R, srMC2R, srMC3R, srMC4R, and srMC5R paralogs, the nucleotide sequences for the *srmcrs* were separately synthesized with a V-5 epitope tag at the N-terminal of the receptor, and inserted into a pcDNA3.1 expression vector (GenScript; Picataway, NJ, USA). Each *srmcr* cDNA was separately transiently transfected into Chinese Hamster Ovary (CHO) cells. The CHO cells were grown at 37 °C in a humidified 5% CO_2_ incubator in DMEM/F12 with 5% fetal calf serum. Each sr cDNA was co-expressed with a CRE/Luciferase reporter plasmid [2] using the Solution T Cell Line Nucleofector Kit (Amaxa Inc., Gaithersburg, MD, USA) and program U-23 [4]. The transiently transfected cells were seeded on a 96-well plate at a density of 1×10^−5^ cells/well. After 48 h in culture, the transfected cells were stimulated with either synthetic srACTH(1-24), srDes-acetyl-α-MSH, srβ-MSH, srγ-MSH, srδ-MSH, srβ-endorphin or hACTH(1-24), or NDP-MSH at concentrations ranging from 10^−6^ M to 10^−12^ M, in serum-free CHO media for four hours at 37 °C. At the end of the incubation period, 100 µl of Bright-Glo luciferase assay reagent (Promega Inc., Madison, WI, USA) was added to each well, and incubated for 5 min at room temperature. Luminescence was measured with a Bio-Tek Synergy HT plate reader (Bio Tek, Winooski, VT, USA), and the dose response curves were analyzed by using Kaleidagraph software (Synergy Software, Reading, PA, USA). All experimental treatments were performed in triplicate.
