# Supplementary material for: Data for amino acid alignment of Japanese stingray melanocortin receptors with other gnathostome melanocortin receptor sequences, and the ligand selectivity of Japanese stingray melanocortin receptors
Source: Data Brief. 2016 Apr 26;7:1670–7. doi: 10.1016/j.dib.2016.04.050 (PMC4927774; doi:10.1016/j.dib.2016.04.050)
Supplement: Supplementary file 1 — Supplementary material [file mmc1.docx]

The authors declare no conflict of interest associated with this manuscript.
